# Targeting Cytochrome P450 Enzymes in Ovarian Cancers: New Approaches to Tumor-Selective Intervention

**DOI:** 10.3390/biomedicines11112898

**Published:** 2023-10-26

**Authors:** Yousef M. Al-saraireh, Fatemah O. F. O. Alshammari, Omar H. Abu-azzam, Sa’ed M. Al-dalain, Yahya M. Al-sarayra, Mansour Haddad, Hafiz Makeen, Aiman Al-Qtaitat, Mohammad Almermesh, Sameeh A. Al-sarayreh

**Affiliations:** 1 Department of Pharmacology, Faculty of Medicine, Mutah University, P.O. Box 7, Al-Karak 61710, Jordan; gregorio@mutah.edu.jo; 2 Department of Medical Lab Technology, Faculty of Health Sciences, The Public Authority for Applied Education and Training, Shuwaikh 15432, Kuwait; fo.alowayed@paaet.edu.kw; 3 Department of Obstetrics and Gynecology, Faculty of Medicine, Mutah University, P.O. Box 7, Al-Karak 61710, Jordan; omar82@mutah.edu.jo; 4 Al-Karak Governmental Hospital, Ministry of Health, P.O. Box 86, Al-Karak 11118, Jordan; yahyasar55@gmail.com; 5 Faculty of Pharmacy, Yarmouk University, Irbid 21163, Jordan; mansour.haddad@yu.edu.jo; 6 Department of Clinical Pharmacy, College of Pharmacy, Jazan University, Jazan P.O. Box 114, Saudi Arabia; hafiz@jazanu.edu.sa; 7 Department of Anatomy and Histology, Faculty of Medicine, Mutah University, P.O. Box 7, Al-Karak 61710, Jordan; aimanaq@mutah.edu.jo; 8 Faculty of Dentistry, Zarqa University, Zarqa 13110, Jordan; 9 Department of Pharmacology, College of Pharmacy, University of Hail, Hail 2440, Saudi Arabia; m.almermesh@uoh.edu.sa; 10 Department of Biochemistry and Molecular Biology, Faculty of Medicine, Mutah University, P.O. Box 7, Al-Karak 61710, Jordan; sameeh_sarayreh@mutah.edu.jo

**Keywords:** cytochrome P450, ovarian cancer, gynecologic cancers, prodrug, xenobiotics, prognosis

## Abstract

Over the past decade, there have been significant developments in treatment for ovarian cancer, yet the lack of targeted therapy with few side effects still represents a major issue. The cytochrome P450 (CYP) enzyme family plays a vital role in the tumorigenesis process and metabolism of drugs and has a negative impact on therapy outcomes. Gaining more insight into CYP expression is crucial to understanding the pathophysiology of ovarian cancer since many isoforms are essential to the metabolism of xenobiotics and steroid hormones, which drive the disease’s development. To the best of our knowledge, no review articles have documented the intratumoral expression of CYPs and their implications in ovarian cancer. Therefore, the purpose of this review is to provide a clear understanding of differential CYP expression in ovarian cancer and its implications for the prognosis of ovarian cancer patients, together with the effects of CYP polymorphisms on chemotherapy metabolism. Finally, we discuss opportunities to exploit metabolic CYP expression for the development of novel therapeutic methods to treat ovarian cancer.

## 1. Introduction

Ovarian cancer is the third most prevalent gynecologic cancer, following uterine and cervical cancers [1]. It develops in the cells of the ovaries or other cells of the fallopian tube and peritoneum. Ovarian cancers are classified as epithelial or non-epithelial, with epithelial being more common. Within this classification, numerous histological ovarian cancer subtypes differ in their pathophysiology and clinical characteristics [2]. Notably, ovarian cancer is recognized to have the worst mortality rate and the poorest prognosis compared to other female cancers. Despite being less common than breast cancer, ovarian cancer is fatal three times more often, and it is estimated that by 2040, its mortality rate will significantly increase [1]. This increase is attributed to the asymptomatic development of tumors, the delayed manifestation of symptoms, and inadequate screening programs that lead to its diagnosis only in the late stages of the disease [1,2]. Despite modern advances in cancer diagnosis technology, the detection rate for invasive ovarian cancer is quite low, and the process is challenging, using a combination approach of transvaginal ultrasound and the serum cancer antigen-125 (CA-125) test. Moreover, the current treatment strategies for ovarian cancer are complicated and have led to only modest improvements in clinical outcomes, particularly in patients with advanced stages of the disease [3]. Studies in pathophysiology, progression, prognosis, and ovarian cancer therapy have been conducted, with researchers showing a particular interest in the implication of metabolic oxidase enzymes in ovarian cancer.

The cytochrome P450 (CYP) superfamily of enzymes comprises 57 human isotypes belonging to the group of self-oxidizing mono-oxygenases. They are categorized into different families, of which the CYP1, CYP2, and CYP3 enzyme families are mainly involved in the metabolism of drugs, with the CYP4 family playing a minor role [4]. These particular families are responsible for detoxifying and inactivating various therapeutic drugs and, most importantly, activating prodrugs into cytotoxic metabolites. The CYP4 to CYP51 enzyme families are mainly involved in the metabolism of endogenous molecules like vitamins, fatty acids, and steroid hormones [5]. On the other hand, CYPs can transform chemical compounds such as nitrosamines into reactive metabolites, triggering organ damage and/or tumorigenesis. For instance, polycyclic aromatic hydrocarbons are converted into phenols, quinones, and catechols by certain members of the CYP1 family, resulting in the generation of diol-epoxides and reactive radical cations, which may interact with DNA to generate DNA adducts. Such DNA adduct formation can lead to mismatches during DNA replication, disrupted promoter methylation, and/or altered promoter binding, resulting in an inherited DNA mutation or aberrant gene expression and, subsequently, carcinogenesis [6]. Moreover, CYP enzymes are susceptible to inhibition and/or induction by a variety of chemicals, which may affect drugs’ therapeutic responses [4,5]. Importantly, the inter-individual variations between CYP isoforms in their gene expression and/or enzymatic activity mainly occur due to differences in genetic alleles or polymorphisms. These variations are the main factors that underlie susceptibility to diseases like cancer and differences in drug pharmacokinetic profiles [5,7]. Although the majority of CYPs are primarily detected in the liver, a few CYP isoforms of certain families are found in extrahepatic tissues. The expression of these extrahepatic enzymes is identified as dysregulated in many organs, possibly contributing to tumorigenesis [8]. Notably, CYP1B1, CYP2W1, CYP2J2, and, more recently, CYP4Z1 have been determined to have cancer-specific expression [9,10,11,12,13,14,15]. Capitalizing on CYP catalytic activity, these cancer-specific CYP enzymes offer novel opportunities for developing selective targeted therapies for cancers expressing these CYP enzymes (Table 1).

The role of CYPs in the tumorigenesis process is of great interest as the expression of certain CYP isoforms has been determined to be altered and possibly associated with the development of ovarian cancer. To the best of our knowledge, no review articles have documented the intratumoral expression of CYPs and its implications in ovarian cancer. Therefore, the current review focuses on current findings on individual CYP expression in ovarian cancer, together with the effects of CYP gene polymorphisms on the metabolism of chemotherapeutic agents and patient prognosis.

## 2. CYPS in Ovarian Cancers

### 2.1. CYP1A1

The induction of CYP1A1 expression is transcriptionally regulated by the activation of cytosolic aryl hydrocarbon receptor (AhR) upon its binding to different ligands. Few studies have investigated CYP1A1 mRNA and protein expression in ovarian cancer cell lines and clinical specimens. Significant levels of CYP1A1 mRNA and protein were detected in the ovarian cancer cell lines A2780 and SKOV-3 relative to their high expression in breast cancer cell lines [16]. Similar results were obtained with four other ovarian cancer cell lines, demonstrating significant overexpression of CYP1A1 mRNA and protein compared to normal ovary cell lines [17].

In clinical specimens, and according to The Cancer Genome Atlas (TCGA), data from 373 patient samples of ovarian cancer associated with CYP1A1 revealed that the mean mRNA expression of CYP1A1 was 0.1 FPKM with a median expression of 0.04 FPKM [18]. Moreover, the CYP1A1 protein was moderately expressed in all examined cases (12 patient samples of ovarian cancer), with its expression confined to the cytoplasm of cells. Based on the aforementioned data, CYP1A1 was not recognized as a prognostic biomarker for ovarian cancer [19]. Similarly, moderate-to-high cytoplasmic expression of CYP1A1 was identified in all patient cases of ovarian cancer compared with ovarian benign epithelia and healthy normal tissues [17]. In contrast, low expression of CYP1A1 comparable to its expression in normal ovary samples was only detected in 20% of ovarian cancer patient samples [20]. These contradictory results may be due to the use of different types of antibodies, which could mistake CYP1A1 for a novel CYP1A1-like enzyme variant called CYP1A1v. This enzymatically active variant was found to be highly expressed in ovarian cancer cell lines and predominantly localized to the cytoplasm and nucleolus [17].

There are no studies exploring the cellular and mechanistic role of CYP1A1 in ovarian cancer. However, evidence of this role has been obtained in investigations of breast cancer. It was shown that the growth and survival of breast cancer cells was impaired upon CYP1A1 knockdown, partly due to the activation of the AMP-activated protein kinase (AMPK) pathway and suppression of the phosphorylation of 70-kDa ribosomal protein S6 kinase (P70S6K), extracellular signal-regulated kinases 1 and 2 (ERK1/2), and AKT [21]. Moreover, CYP1A1 was found to play a critical role in breast cancer stem cell integrity, whereby it controlled cell growth, self-renewal, and chemoresistance, presumably via catenin and PTEN/AKT signaling [22]. Importantly, proof-of-concept studies utilizing CYP1A1 for the development of cancer-targeted therapies have been conducted by many groups (Table 1). The investigation by Shnyder and co-workers on re-engineering duocarmycin molecules represents one example of prodrug strategies relying on tumor-expressed CYP1A1 for bioactivation [23]. CYP1A1 has also been found to be a targetable enzyme in cancer cell line models and xenograft studies of colon and bladder cancers [24,25]. Another group also reported the development of CYP1A1-targeting prodrugs of anti-mitotics, which exert potent cytotoxicity in both in vitro and in vivo models of breast cancer [26].

### 2.2. CYP1B1

CYP1B1 is an extrahepatic enzyme belonging to the CYP1 family and shares about 40% homology with other members, including CYP1A1 and CYP1A2. Similar to CYP1A enzymes, the induction of CYP1B1 expression is mainly regulated via the activation of AhR receptors by different ligands like dioxins and polycyclic hydrocarbons [4]. In ovarian cancer cell lines, high expression of CYP1B1 mRNA and protein was detected in A2780, SKOV-3, OVCA 420, OVCA 429, OVCA 432, and OVCA 433 [16,17]. Based on data from The Human Protein Atlas, among 180 samples of normal ovaries, the average CYP1B1 mRNA expression was 5.6 nTPM with a median expression of 2.6 nTPM, while CYP1B1 protein expression was low [19]. These results are consistent with previous studies reporting the absence or low protein expression of CYP1B1 in normal ovary tissues [20,27]. Importantly, among 373 patient samples of ovarian cancers, TCGA showed that the average CYP1B1 mRNA expression was 4.6 FPKM, with a median expression of 2.86 FPKM, while moderate-to-high CYP1B1 protein expression was detected [18,19]. Consistently, McFadyen et al. investigated CYP1B1 expression in 172 patient samples of primary and metastatic ovarian cancers. Elevated CYP1B1 expression was determined in 92% of the samples and frequently localized to the cytoplasm of cells with no obvious heterogeneity [20]. Similarly, a significantly higher level of CYP1B1 expression was seen in almost 90% and 70% of primary and metastatic ovarian cancers, respectively [27]. Importantly, neither CYP1B1 mRNA expression nor protein expression proved to be biomarkers of prognosis [18,20].

Several studies have investigated the molecular mechanisms underlining CYP1B1-mediated tumorigenesis [28,29,30,31]. It was demonstrated that wild-type mice with CYP1B1 developed higher rates of cancer in many organs (including the ovaries) than CYP1B1-null mice following treatment with a chemical carcinogen. Moreover, the DNA adducts in wild-type mice and V79 cells expressing CYP1B1 were higher than those in CYP1B1-null mice and CYP1B1-deficient V79 cells [28]. Additionally, CYP1B1 depletion was shown to reduce the growth, invasion, and migration of cancer cells through the upregulation of cell division cycle 20 homolog (CDC20) and down-regulation of death-associated protein kinase-1 (DAPK1) [29]. CYP1B1 was also found to enhance cancer progression through the induction of the epithelial–mesenchymal transition (EMT) and the Wnt/β-catenin pathway through Sp1 induction [30]. Furthermore, increased CYP1B1 expression was correlated with higher drug resistance in breast and ovarian cancer cells [32,33,34]. Recently, CYP1B1 was also shown to enhance the resistance of cancer cells to ferroptosis and immunotherapy, particularly anti-PD-1, through the activation of the protein kinase C (PKC) cascade and the degradation of acyl-CoA synthetase long-chain family member 4 (ACSL4) [31]. Such results support CYP1B1’s significance as a marker of drug resistance and a prognostic predictor for therapy.

### 2.3. CYP2A6 and CYP2B6

Since CYP2A6 plays a major role in nicotine metabolism, it is implicated in tobacco-related diseases like lung cancer. Additionally, it is one of the numerous CYPs that is primarily engaged in the metabolism of drugs like coumarin, halothane, and tamoxifen. This enzyme is liver-specific but displays extrahepatic expression in some organs [4]. According to TCGA data, there is low CYP2A2 mRNA expression and an absence of protein expression in samples of normal ovaries and ovarian cancer. For the CYP2B6 enzyme, the expression of mRNA and protein was detected in neither normal ovary nor ovarian cancer tissues [18,19]. These data contrast with earlier studies reporting significant expression of CYP2A/2B enzymes in ovarian cancer tissues compared to normal ovary tissues [20]. One possible explanation for these conflicting results is that the antibody used for the detection of CYP2A6 cross-reacts with CYP2A6 and CYP2B6 enzymes, as both enzymes have nearly identical COOH-terminal amino acid sequences.

### 2.4. CYP2C8

The CYP2C subfamily comprises four closely homologous enzyme genes: CYP2C8, CYP2C9, CYP2C18 and CYP2C19. These enzymes are mainly expressed in the human liver. The trifurcated, large active site of CYP2C8 resembles that of CYP3A4 and is different from other CYP2C family members, allowing it to handle substrates of various sizes and shapes [35]. CYP2C8 is transcriptionally regulated by several factors and diverse nuclear receptors activating necessary elements within the gene’s 59-flanking promoter region. These factors and/or receptors include, but are not limited to, constitutive androstane receptor (CAR), glucocorticoid receptor (GR), pregnane X receptor (PXR), hepatic nuclear factor-4a (HNF4a), and vitamin D receptor (VDR) [36]. CYP2C8 is widely recognized as participating in the metabolic biotransformation of endogenous compounds, including over 100 different drugs, including, but not limited to, anticancer drugs. CYP2C8 has been identified in many extrahepatic tissues, including the heart, kidneys, salivary ducts, tonsils, adrenal cortical cells, and small and large intestines [35]. Although low levels of CYP2C8 mRNA were found in normal ovaries [37], no evidence of protein expression was given. However, CYP2C8 mRNA and protein were detected in primary ovarian cancer, and albite protein expression was weak [18,19]. These findings are consistent with those of other studies demonstrating CYP2C8 expression in most of the ovarian cancer samples examined [20,33].

### 2.5. CYP2C9

CYP2C9 is an epoxygenase enzyme that metabolizes several endogenous compounds (like arachidonic acid) and xenobiotics (like anticancer drugs). It is the second most abundant CYP expressed in liver cells after CYP3A4 [4,38]. About 15% to 20% of all drugs that pass phase I metabolism are metabolized by CYP2C9. Furthermore, CYP2C9 expression was shown to be induced by rifampicin treatment [4]. CYP2C9 expression has been identified in numerous organs, where it is expressed differently in non-neoplastic and malignant human tissues. However, the expression of CYP2C9 mRNA and protein has not been detected in either normal ovaries or ovarian cancer [9,18,39].

### 2.6. CYP2E1

In addition to being a phase 1 drug-metabolizing enzyme, CYP2E1 is linked to a number of diseases, including cancer, type 2 diabetes, obesity, and liver disorders caused by alcohol intake [40]. While there are few investigations on the expression of CYP2E1, the available data reveal comparable CYP2E1 expression patterns were found at both the mRNA and protein levels in normal ovaries [19,20]. Evidence of CYP2E1 protein expression is only available from one study reporting non-significant protein expression in primary and metastatic ovarian cancers compared to normal ovaries [20]. Importantly, a marked increase in CYP2E1 activity was found in the sera of ovarian cancer patients. Such increased activity is associated with tumor-induced inflammation exhibited by high serum levels of proinflammatory cytokines like TNF-α, IL-6, and IL-8 [41].

### 2.7. CYP2J2

CYP2J2 is an arachidonic acid epoxygenase enzyme that is regulated via the activation of microRNA let-7b, protein-1 (AP-1), and the AP-1-like element. It is mainly involved in the metabolism of arachidonic acid, generating four isomers of epoxyeicosatrienoic acids (EETs) [42]. Several lines of evidence highlight the potential role of CYP2J2 and its mediated products in the cancer pathogenesis of many human tumors [43]. In normal ovaries, the expression of CYP2J2 mRNA and protein expression has not been identified [18,19]. However, high expression of CYP2J2 mRNA has been found in a large patient cohort of primary and malignant ovarian cancers compared to that in normal and benign tumors of the ovary [18,39]. Evidence of CYP2J2 protein expression is only available from one study reporting non-significant protein expression in ovarian cancers compared to normal ovaries [18]. Mechanistic studies demonstrated that high CYP2J2 expression strongly enhanced proliferation and reduced apoptosis in several cancers via the activation of kinase/Akt signaling pathways and increased the phosphorylation of epidermal growth factor receptor (EGFR). All of these effects were reversed in both in vitro and in vivo models via treatment with CYP2J2 inhibitors, implying that CYP2J2 provides cancer cells with a protective mechanism to increase their survival [44,45].

### 2.8. CYP2S1

CYP2S1 is an orphan CYP enzyme displaying many characteristics that are typical of enzymes of the CYP1 family. It shares dioxin-inducibility catalyzed by AhR, suggesting a potential role in both exogenous and endogenous compound metabolism. It is primarily involved in the formation and metabolism of lipids such as prostaglandins and retinoids [46]. Although studies on the expression of CYP2S1 are limited, the available data reveal that comparable CYP2S1 expression patterns were determined at both the mRNA and protein levels in normal ovaries [20,46]. These results are consistent with data from The Human Protein Atlas, showing a similar pattern of expression of CYP2S1 mRNA and protein in normal ovaries [19]. Moreover, CYP2S1 was found to be more highly expressed in ovarian cancers than in healthy normal ovaries, and significant CYP2S1 expression was identified in metastatic ovarian tumors compared to primary ovarian cancers and healthy normal ovaries [20,46]. The implications of this finding remain to be determined. Regarding the functional role of CYP2S1 in the tumorigenesis process, Guo and co-workers reported that CYP2S1 knockdown reduced the proliferation, invasion, and migration of lung cancer cells. Moreover, the inhibition of CYP2S1 expression in animal models decreased the growth of lung cancer [47].

### 2.9. CYP2U1

CYP2U1 is a hydroxylase enzyme implicated in the metabolism of fatty acids like arachidonic acid and N-arachidonoylserine. The up-regulation of CYP2U1 is induced by glutamate through the phosphorylation of cAMP-response element binding (CREB) proteins and the binding of these phosphorylated proteins with the CYP2U1 promoter in the nucleus [48]. Investigations have revealed low expression of CYP2U1 mRNA and protein in normal ovaries [18,20,49]. These data are in contrast with an earlier study reporting high levels of CYP2U1 mRNA in normal ovaries [50]. However, moderate expression of CYP2U1 mRNA and protein was identified in ovarian cancers. Concurrently, a significant differential in the expression of the CYP2U1 enzyme was found, and its expression was high in primary and metastatic ovarian cancers compared to normal ovaries [20].

### 2.10. CYP3A4

CYP3A4 is the most abundant CYP found in the adult human liver, making up about 30% of the overall CYP protein level. It is also found in high concentrations in the colon, small bowel, and pancreas [4,19]. CYP3A4 plays an essential role in the activation and detoxification of a wide range of xenobiotics and endogenous compounds. It is involved in the activation of several procarcinogens like aflatoxins, polycyclic hydrocarbon dihydrodiols, and heterocyclic amines. Importantly, CYP3A4 is also involved in the metabolism of 60% of pharmaceutical drugs, together with chemotherapy used for ovarian cancer treatment, such as docetaxel and paclitaxel [4,51]. Furthermore, CYP3A4 was shown to convert testosterone and estrogen to several metabolites that play a major role in the etiology of ovarian and breast cancers [52]. Studies of cancers have found differential CYP3A4 expression in breast, esophageal, colorectal, and Ewing’s sarcoma tumors compared to matched normal tissues [18,19]. The available data on the expression of CYP3A4 mRNA and protein in normal and cancerous ovarian tissues are contradictory. CYP3A4 mRNA was found at basal levels in normal ovaries, whereas CYP3A4 protein was not detected. Similarly, extremely low levels of CYP3A4 mRNA were identified in primary ovarian cancer samples, while no CYP3A4 protein was expressed. These findings are consistent with DeLoia et al.’s study reporting rare and extremely low CYP3A4 mRNA expression in ovarian cancer [33]. In contrast, high and more frequent CYP3A4 protein expression was found in both normal ovaries and primary ovarian cancer, although its differential expression was not significant [20].

### 2.11. CYP3A5

CYP3A5 is a mono-oxygenase enzyme found to be expressed in hepatic and extrahepatic organ tissues. This enzyme is involved in the production of steroids and lipids and the metabolism of many drugs, such as anticancer drugs. Although the CYP3A5 enzyme’s substrate specificity is similar to that of CYP3A4, CYP3A5 is considered less crucial for drug elimination due to its substantially lower expression than CYP3A4 in adult livers [53]. Compelling data on the expression of CYP3A5 mRNA and protein in normal and cancerous ovary tissues are available. Data from The Human Protein Atlas show that healthy ovary tissues express basal levels of CYP3A5 mRNA [19]. In contrast, Bièche et al. found high levels of CYP3A5 mRNA in normal ovaries [50]. Importantly, CYP3A5 protein was found to be less frequently expressed in normal ovaries and significantly expressed in primary and metastatic ovarian cancers. However, this significant expression did not prove to be a prognostic marker for ovarian cancers [18,20,33].

### 2.12. CYP3A7

CYP3A7 is a fetal hepatic enzyme that shares about 93% homology with CYP3A4. During embryonic development, CYP3A7 is mainly involved in estriol biosynthesis, all-trans retinoic acid clearance, and xenobiotic metabolism [54]. While CYP3A7 is mainly expressed in the fetal liver, it is also available in some adult livers, albeit at low levels compared to CYP3A4. It is also found in fetus extrahepatic tissues such as the intestines, placenta, endometrium, prostate, adrenal gland, and lungs [55]. However, quantitative data on CYP3A7 expression in adult hepatic and extrahepatic tissues, particularly normal ovaries, are limited. CYP3A7 mRNA expression was less frequently found in normal ovaries [19]. Meanwhile, a similar pattern of expression was found in a large cohort of ovarian cancers [18]. Evidence of CYP3A7 protein expression was obtained in a study demonstrating that CYP3A7 protein was significantly expressed in both primary and metastatic ovarian cancers compared to its low levels of expression in normal ovaries [20]. This finding contradicts the data on CYP3A7 mRNA expression in ovarian cancers. One explanation for this finding may arise from the specificity of the antibody used to detect CYP3A7 protein expression, as CYP3A4-to-CYP3A7 cross-reactivity may occur.

### 2.13. CYP3A43

CYP3A43 is a hydroxylase enzyme and represents the least abundant and characterized member of the CYP3 family. Its amino acid sequence shares about 76%, 76%, and 72% homology with CYP3A4, CYP3A5, and CYP3A7, respectively [56]. To date, only dexamethasone and rifampicin have been found to induce the expression of CYP3A43. Moreover, recent data indicated the potential role of CYP3A43 in metabolizing endogenous and xenobiotic compounds like other CYP3 members, albeit to a lesser extent. CYP3A43 was found to participate in the biotransformation of endogenous testosterone and some drugs, including alprazolam and olanzapine. CYP3A43 is primarily expressed in the prostate but is also present at relatively low levels in the hepatic tissues compared to other CYP3 members [57]. The available data on CYP3A43 expression patterns in normal and cancerous ovary tissues are controversial. Data from The Human Protein Atlas show that neither CYP3A43 mRNA nor CYP3A43 protein was detected in normal ovaries [18]. Moreover, TCGA data on ovarian cancers demonstrate that CYP3A43 mRNA was identified at extremely low levels, while the protein was not detected at all [18]. On the other hand, CYP3A43 protein was found to be more frequently expressed in primary and metastatic ovarian cancers. Significant differences in the intensity of CYP3A43 protein expression between primary ovarian cancer and normal ovary samples were of particular interest [20]. Recently, CYP3A43 expression was found to play an unprecedented role in the tumorigenesis process, particularly in lung adenocarcinoma. CYP3A43 depletion promoted the proliferation and migration of lung cancer cells and enhanced xenograft lung cancer growth. Interestingly, CYP3A43 overexpression decreased cell proliferation and did not alter cell migration or colony formation. Further studies are required to clarify this unprecedented role of CYP3A43 [58].

### 2.14. CYP4B1

CYP4B1 is an omega-hydroxylase orphan enzyme and is the only known member of the CYP4B family. CYP4B1 expression was found to be induced by androgens and down-regulated by soy isoflavones [59,60]. This pattern of expression was regulated by multiple nuclear receptors, like nuclear factor-kappa light chain enhancer of activated B cells (NF-kB), activator protein 1, hypoxia-inducible factor 1 (HIF-1), retinoid X receptor (RXR), and AhR [5]. Additionally, CYP4B1 is predominantly expressed in human lungs and other human organs, albeit at extremely low levels [5,61]. In the carcinogenesis process, it was found to play an important role in neovascularization and procarcinogen activation. Recent data show that CYP4B1 gene expression was identified in cancers of the lung, liver, bladder, prostate, breast, and ovaries [5,61]. In ovarian cancers, patients with recurrent serous ovarian cancer were found to have higher CYP4B1 mRNA than patients with cured ovarian cancer. In contrast, only 8.3% of patients with ovarian cancers displayed medium CYP4B1 protein expression [18,62]. In normal ovaries, low levels of CYP4B1 mRNA were found, while no protein expression was seen [19].

### 2.15. CYP4Z1

CYP4Z1 is an orphan enzyme that exhibits fatty acid hydroxylase or epoxygenase catalytic activity. The induction of CYP4Z1 mRNA is conditionally regulated by progesterone and glucocorticoids and blocked by the steroid receptor inhibitor mifepristone [63]. CYP4Z1 exhibits both hydroxylase and epoxygenase catalytic activity; in particular, it converts mid-chain fatty acids (lauric and myristic acids) to their monohydroxylated derivatives and arachidonic acid to 20-hydroxyeicosatetraenoic acid (20-HETE) or 14,15-epoxyeicosatrienoic acid (14,15-EET), respectively [64,65]. CYP4Z1 mRNA expression is preferentially found in the mammary glands, but lower levels are detected in the heart, liver, brain, kidneys, prostate, testes, lungs, and ovaries [11,66]. Clinical investigations into CYP4Z1 protein’s expression profile in cancers reveal a promising trend: a significant difference in CYP4Z1 protein expression between many cancers and their corresponding normal tissues, such as breast, lung, prostate, bladder, colon, cervix, and recently, ovary tissues. Importantly, CYP4Z1 expression was found to be associated with high-grade and late-stage disease and proved to be a poor prognostic marker for these cancers [9,11,12,13,14,15,20,67]. Interestingly, CYP4Z1 expression was found to stimulate the generation of CYP4Z1 autoantibodies in the sera of patients with cancers of the breast, lung, colon, prostate, and ovaries. However, no significant difference was found in the levels of CYP4Z1 autoantibodies generated in the sera of these cancer patients compared to normal controls [68,69]. A possible explanation for this finding is that the sample sizes in these studies were not large enough to demonstrate such differences.

Despite the lack of functional research examining CYP4Z1’s mechanistic role in the development of ovarian cancer, various investigations have connected CYP4Z1 to the tumorigenesis process [70,71,72]. CYP4Z1 was reported to strongly promote tumor growth, metastasis, and neovascularization in cell lines and animal models. Compared to control cells, CYP4Z1 overexpression in tumor cells increased the expression levels of vascular endothelial growth factor A (VEGF-A) and suppressed the expression levels of tissue inhibitors of metalloproteinases 2 (TIMP-2) [70]. Additionally, elevated levels of 20-HETE and low levels of lauric and myristic acids were also produced [64,70]. Moreover, CYP4Z1 expression was found to enhance cancer cell stemness and tamoxifen drug resistance [72]. Another investigation revealed that CYP4Z2P and its pseudogene, CYP4Z1-3′UTR, enhanced tumor neovascularization in breast cancer, presumably by activating the ERK1/2 and PI3K/Akt cascades [71]. 

### 2.16. CYP26A1

CYP26A1 is a highly conserved member of the CYP26 family, along with CYP26B1 and CYP26C1. CYP26A1 works by metabolizing and eliminating all-trans retinoic acid (ATRA), a bioactive molecule of retinol or vitamin A that is implicated in the regulation of cellular differentiation, migration, proliferation, and death [73]. CYP26A1 overexpression was found to trigger cell survival and antiapoptotic pathways through the downregulation of tumor suppressor genes and the induction of oncogenes [73,74]. The main function of CYP26A1 seems to be in the biotransformation of retinoic acid (RA) to its primary metabolite, 4-OH-RA, along with other minor metabolites. Moreover, CYP26A1 is also involved in the metabolism of exogenous compounds like tazarotenic acid [74,75]. CYP26A1 mRNA has predominantly been found in the human liver, with smaller amounts found in other organs, including the lungs, kidneys, testes, and skin. In normal ovaries, CYP26A1 mRNA was not detected in [19], although low CYP26A1 protein expression was exhibited in less than 10% of samples examined in [20]. Importantly, comparable levels of CYP26A1 mRNA were found in ovarian cancers [18]. Consistent with this, significant high CYP26A1 protein expression was displayed in primary ovarian cancers, along with intense expression in metastatic ones [20].

### 2.17. CYP51A1

CYP51A1 is a 14α-demethylase enzyme that plays an important role in the biosynthesis of cholesterol. It is a highly conserved enzyme that possesses about 95% amino acid sequence identity among mammals [76]. CYP51A1 selectively catalyzes lanosterol and 24,25-dihydrolanosterol via alpha-demethylation, forming the other sterols necessary for cholesterol biosynthesis. CYP51A1 mRNA is widely distributed throughout the human body tissues, although the highest levels were found to be present in testes [77]. Limited data are available on the expression of CYP51A1 mRNA and protein in normal and cancerous ovary tissues. It was shown that comparable levels of CYP51A1 mRNA were found in normal ovaries, while no protein expression was detected [78]. A similar pattern of expression for CYP51A1 mRNA was also displayed in ovarian cancers, though low protein expression was detected in less than 10% of the ovarian cancer samples examined [18]. In contrast, CYP51A1 protein was significantly expressed in almost half of patients with primary ovarian cancer compared to its weak expression in almost 10% of normal ovary samples. Moreover, CYP51A1 protein expression was displayed in almost 20% of metastatic ovarian cancer samples [20].

## 3. Impact of CYP Polymorphisms on Risk and Prognosis of Ovarian Cancer

**Figure 1:**
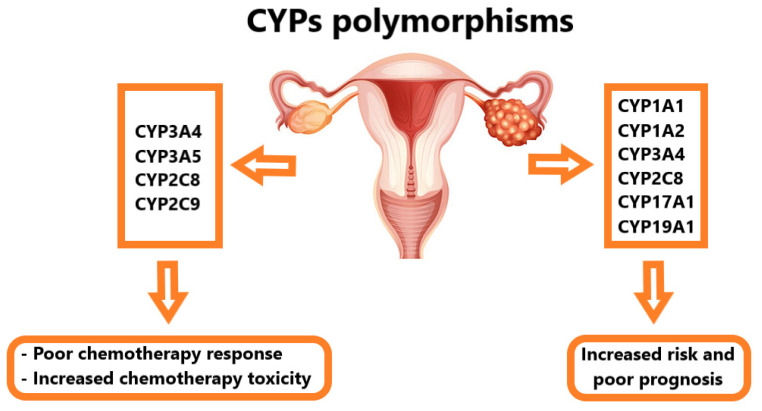
The effects of CYP polymorphisms on ovarian cancer prognosis and therapy.

In light of the importance of CYPs in the biotransformation of precarcinogens, few studies have attempted to identify genetic variants of each CYP that may predispose patients to ovarian cancer (Figure 1). Several studies have examined the association between the genotype status of the most common CYP1A1 polymorphisms (Ile462Val, I462V, MspI, M4, and Thr461Asn) and the risk of ovarian cancer development [79,80,81,82,83]. The risk of developing ovarian cancer increased among patients with genetic variants of CYP1A1 Ile462Val, I462V, MspI, and M4 [79,80,81,82,83,84]. No association of ovarian cancer risk was found with CYP1A1*4 (Thr461Asn) alleles [83]. Moreover, a positive association of CYP1A2 genetic variants with ovarian cancer risk was detected in patients who were tobacco smokers or coffee drinkers. No relevant associations of CYP1A2 genetic variants with ovarian cancer risk were found in non-smokers or non-coffee drinkers [81,85]. In contrast, the A allele of the CYP1A2*1F genetic variant was found to decrease the risk of ovarian cancer [86]. Regarding CYP1B1, all studies, except for Zhang’s, found that polymorphisms in the CYP1B1 gene were not significantly associated with ovarian cancer susceptibility [87,88,89,90]. Here, the genetic variant of CYP1B1 rs1056836 showed a significant association with ovarian cancer susceptibility among Asians and Caucasians, while no association was detected among African Americans [90].

Additionally, reports have shown that the CYP3A4 rs2740574 variant is inversely associated with ovarian cancer morbidity [91,92]. These data contradict earlier studies indicating the absence of an association between the CYP3A4 rs2740574 variant and ovarian cancer risk [93,94]. Other CYP3A4 genetic variants had no impact on ovarian cancer susceptibility [94]. Similarly, the difference in the CYP17 genetic polymorphism of T→C between ovarian cancer cases and controls was not significant; therefore, this genetic variant did not affect ovarian cancer risk [95]. However, in a recent study, the genetic variant of CYP17A1 (rs743572) showed a significant association with ovarian cancer risk [96]. Regarding CYP19, the risk of ovarian cancer increased among carriers of one or both genetic variants of CYP19013 A or CYP19027 G [97]. Moreover, female carriers with CYP19 gene polymorphisms of (TTTA) 11 and (TTTA) 12 were at a twofold and fourfold increased risk of developing ovarian cancer, respectively [98]. Recently, the rs10046 (CYP19A1) variant was found to be associated with an increased risk of developing ovarian cancer [96]. Regarding the CYP2C8 enzyme, patients carrying the CYP2C8*1D variant enzyme had poor prognoses following chemotherapy treatment [99]. Considering the apparent scarcity of investigations and their limited sample sizes, studies on the polymorphisms of the CYP2D6, CYP2E1, and CYP3A5 genes demonstrate a lack of genotype associations with ovarian cancer susceptibility [94,100,101,102,103]. In summary, no significant conclusions can be drawn from these observational studies. This is largely because of the limited number of studies on this topic and their recruitment of small numbers of cases and controls, with improper confounding factor control and a lack of information regarding CYP gene interactions with some other genes and/or external environmental factors.

## 4. Impact of CYP Polymorphisms on Chemotherapeutic Agent Metabolism in Ovarian Cancer

The pharmacological response of chemotherapeutic drugs in patients with ovarian cancer is greatly influenced by CYP genetic polymorphisms (Figure 1). They trigger changes in drug response varying from adverse drug reactions to the absence of clinical efficacy. Many anticancer drugs, such as cyclophosphamide, docetaxel, paclitaxel, 5-fluorouracil, doxorubicin, and cisplatin, are metabolized by CYP1B1 [104]. However, the genetic polymorphisms of CYP1B1 had no association with patient outcomes, chemotherapy toxicity, or chemotherapy resistance in ovarian cancers [51,90]. Regarding CYP3A4 polymorphisms, compared with patients carrying AA, the risk of disease progression and chemotherapy resistance following treatment with platinum-based drugs was elevated in patients carrying CYP3A4 rs2740574 with at least one G allele [105]. Moreover, CYP3A4 rs2740574 was associated with worse overall survival in patients with ovarian cancer treated with platinum and taxane drugs [92]. Additionally, the mechanism of paclitaxel metabolism was altered by CYP3A4 rs2740574 activity in vivo, but overall paclitaxel clearance was not [106]. In contrast, the CYP3A4*16 genetic allele did not affect paclitaxel pharmacokinetics or metabolism [107]. Despite the strong structural similarity (85% homology) between CYP3A4 and CYP3A5, CYP3A5 polymorphisms had no association with overall survival, patient outcomes, or chemotherapy toxicity in ovarian cancer patients treated with platinum and taxane drugs [51,94,105].

In large cohorts of patients with ovarian cancer treated with carboplatin and either paclitaxel or docetaxel, CYP2C8 gene polymorphisms had no impact on patient outcome and chemotherapy-induced toxicity [51]. Similarly, Gagno et al. found no relationship between CYP2C8 polymorphisms and overall survival, the progression of the disease, or platinum sensitivity in ovarian cancer patients treated with platinum regimens [105]. Other studies highlight the importance of CYP2C8 polymorphisms, particularly the CYP2C8*3 genotype, in the pharmacokinetics of paclitaxel. Ovarian cancer patients carrying the CYP2C8*3 genotype had a decreased clearance rate of unbound paclitaxel compared to wild-type patients [106,108]. Regarding CYP2C9 polymorphisms, this particular genotype of CYP2C9 rs1057910 was associated with a reduced response rate, worse progression of the disease, and a lower overall survival rate in ovarian cancer patients treated with platinum drugs [105]. Additionally, CYP genetic polymorphisms are also strongly correlated with chemotherapy-induced adverse hematological effects in patients with ovarian cancer. Patients carrying CYP3A5*3/*1 genotypes had a significant association with carboplatin/paclitaxel-induced neutropenia and leukopenia compared to control patients [109,110]. Consistent with this, the CYP2C8-HapC genetic variant was shown to significantly induce neutropenia and leukopenia in ovarian cancer patients treated with carboplatin/paclitaxel regimens [109]. Moreover, the CYP2C8*3 polymorphism was found to induce myelosuppression and motor neuropathy in ovarian cancer patients treated with a paclitaxel regimen [106]. Additionally, CYP2C8*1D was found to be a risk factor for the early onset of neurotoxicity following paclitaxel treatment [99]. This may explain the dissatisfactory therapeutic outcomes in certain patients with ovarian cancer. CYP polymorphisms seem to play a relevant role in chemotherapy metabolism in ovarian cancer; however, the current evidence from the small number of studies summarized above remains conflicting and limited.

## 5. Implications of CYP Isoform Expression for Patient Survival

**Figure 2:**
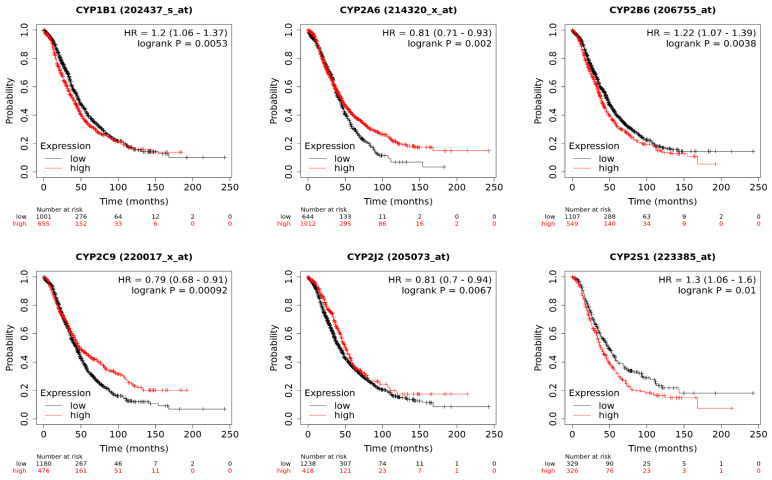

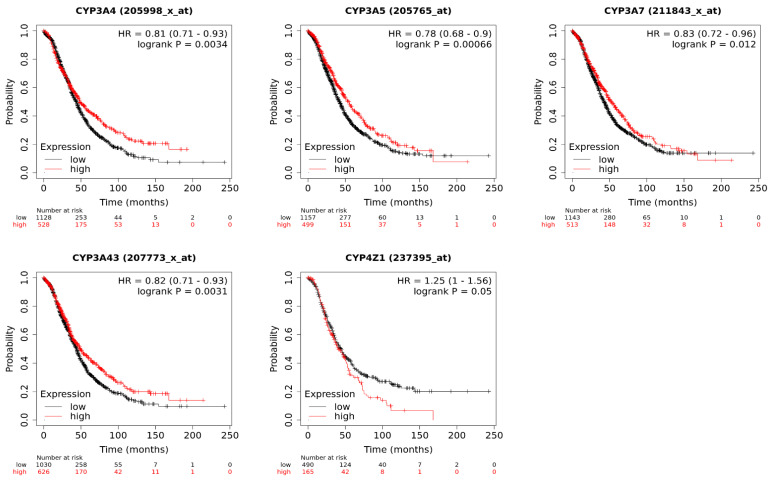
The effect of CYP mRNA expression on ovarian cancer survival according to the Kaplan–Meier analysis. The result is considered significant if *p* ≤ 0.05.

Our KM plotter database analysis of the CYP genes implicated in tumorigenesis and chemotherapy metabolism in ovarian cancer revealed an association of several isoforms with overall patient survival. We determined the mRNA expression of 17 CYP genes, with 11 genes exhibiting significant expression and 6 not exhibiting significant expression in terms of patient survival (Figure 2). High mRNA expression of CYP1B1, CYP2B6, CYP2S1, and CYP4Z1 was significantly associated with poor overall patient survival. In contrast, high mRNA expression of CYP2A6, CYP2C9, CYP2J2, CYP3A4, CYP3A5, CYP3A7, and CYP3A43 connoted a good prognosis for ovarian cancer patients. Other CYP isoforms showed no significant association. Higher expression of CYP3 enzymes has been shown to have protective effects against many cancers by suppressing cancer progression and metastasis [111,112]. Moreover, lower expression of CYP3 enzymes, particularly CYP3A4, may influence the survival of ovarian cancer patients as they may be ineffective in metabolizing or activating cyclophosphamide and doxorubicin, the standard drugs used for the treatment of ovarian cancers [113].

## 6. Association of CYP Isoform Expression with Immune Infiltration

**Figure 3:**
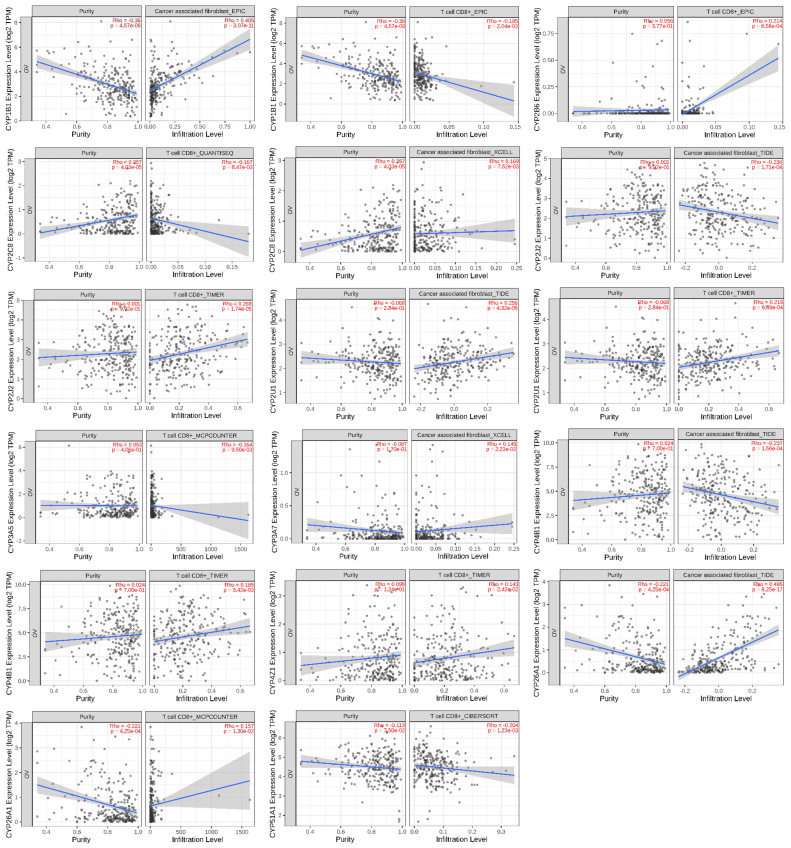
Correlation of CYP enzyme expression with immune infiltration of CAFs and CD8 + T-cells. Blue line indicates regression trend.

One of the main and most significant components of the tumor microenvironment is infiltrating immune cells, which are typically connected with cancer behavior, treatment resistance, and patient prognosis. A growing body of research has shown that cancer-associated fibroblasts (CAFs) are the most common types of cells in the tumor stroma, where they largely help tumor cells escape the immune system by modifying infiltrating immune cells [114]. Moreover, host CD8 + T lymphocytes are crucial for the development, growth, and spread of cancers; their malfunction in cancers is linked to a poor therapeutic response [115]. Hence, the TIMER 2.0 web tool was utilized to evaluate the correlation between the expression of different CYPs and the level of infiltration of immune cells in ovarian cancer. Our results showed that CYP1B1, CYP2C8, CYP2U1, CYP3A7, and CYP26A1 were significantly positively correlated with the infiltration level of CAFs (Figure 3). In contrast, CYP2J2 and CYP4B1 were significantly negatively correlated with the infiltration level of CAFs. Additionally, a statistically positive correlation was found between the infiltration level of CD8 + T-cells and the expression of CYP2B6, CYP2J2, CYP2U1, CYP4B1, CYP4Z1, and CYP26A1. In contrast, a significant negative correlation was found between the infiltration level of CD8 + T-cells and the expression of CYP1B1, CYP2C8, CYP3A5, and CYP51A1. The other types of CYPs studied showed no correlation with the infiltration level of CAFs and CD8 + T-cells. These results may explain the disappointing clinical activity of immune checkpoint inhibitors in the treatment of ovarian cancer [116]. Recently, a link between the expression of CYPs and resistance to immune checkpoint inhibitors in the treatment of cancers was identified [31,117]. It was found that CYP1B1 expression promoted colon cancer cells’ resistance to ferroptosis and induced resistance to anti-PD-1 therapy. The mechanism behind such resistance was attributed to CYP1B1’s capability to metabolize arachidonic acid (AA) to 20-HETE, where 20-HETE activates the PKC pathway, resulting in ACSL4 degradation. Moreover, the inhibition of CYP1B1 expression in cancer cells enhanced treatment efficacy and overcame cancer cells’ resistance to anti-PD-1 therapy [31]. It is well known that CYP1B1 is highly expressed in ovarian cancer and is associated with poor prognosis and anticancer drug resistance [20,27,32,33]. We believe that such resistance to immune checkpoint inhibitors may be mirrored in ovarian cancer and that CYP1B1 inhibitors may play a role in improving and overcoming resistance to immune checkpoint inhibitors.

## 7. Future Perspectives

**Figure 4:**
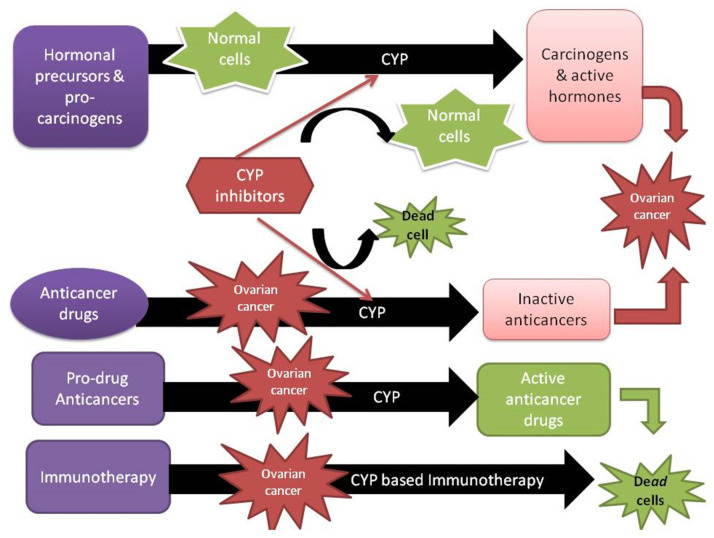
The proposed mechanism and targeted pathway for CYP-directed therapies.

Despite the major developments in treatment options over the last decade, the development of selective ovarian cancer treatment with limited adverse effects remains a significant challenge. Therefore, the individual expression of CYPs in ovarian cancers may represent a potential therapeutic target for the development of specific diagnostic biomarkers and selective tumor-directed therapies. This can be achieved through a variety of therapeutic approaches, such as CYP-based prodrug activation, CYP-targeted immunotherapy, and CYP inhibitors (Figure 4) (Table 1). CYP-based prodrug activation has significant potential for providing substantial therapeutic advantages while resolving the issues related to toxicity that limit the administration of many current anticancer drugs. Examples of such strategies include CPA, tegafur, and tamoxifen. However, the administration of these agents is accompanied by side effects because the bioactivation of these prodrugs is not tumor-selective [118]. Although exciting research in this field has been reported, many prodrug approaches targeting the selective tumor expression of specific CYPs have not yet resulted in drugs being approved for clinical use.

The development of CYP inhibitors for cancer therapy has already made significant progress. For instance, aromatase inhibitors (CYP19 inhibitors) have altered the course of treatment of estrogen-dependent cancers such as breast cancer. This achievement has led to opportunities for similar approaches that inhibit androgen synthesis to treat prostate cancer and possibly ovarian cancer. Molecular entities designed to inhibit the CYP24 and CYP26 that control vitamin D (1.25-D3) and vitamin A (all-trans-retinoic acid; ATRA) metabolism provide advantages in vitamin therapy for the treatment of various cancers, including ovarian. Moreover, the inhibition of xenobiotic metabolizers like CYP1A1 and CYP1B1 by small molecules may have therapeutic benefits because of their potential roles in activating carcinogens and deactivating chemotherapeutics [119].

Among the CYPs expressed in ovarian cancer, only a few overexpressed enzymes have the potential for use in drugs to treat ovarian cancer. Several CYP1B1 cancer treatment strategies are being developed, such as prodrug activation, inhibitors, and immunotherapy. Some of the anticancer prodrugs being examined for CYP1B1 activation include aryl oximes, resveratrol, and phortress [118]. Moreover, CYP1B1 inhibitors like piceatannol aim to promote efficacy and modulate the cytotoxicity of some types of chemotherapeutic agents [120]. Additionally, the CYP1B1 vaccine ZYC300 is an immunotherapy strategy that aims to kill cancer cells expressing CYP1B1 by inducing T-cell activity [118]. All of these CYP1B1-based cancer therapies could unlock new options for the treatment of ovarian cancer.

Of particular importance is the overexpression of the orphan enzyme CYP4Z1 in ovarian cancer [15,20]. Its distinct expression pattern in ovarian cancer makes it an attractive candidate for CYP4Z1-directed therapies. Given the vast experimentation with various human CYPs as prodrug activators, the discovery of suitable candidate CYP4Z1-activated prodrugs is plausible. Once these prodrugs are available, such compounds can be examined in xenograft models of ovarian cancer or other positive-CYP4Z1 cancers, as high levels of CYP4Z1 are abundant in the target tissue, and a gene delivery system is not needed. Nevertheless, one major challenge in the discovery of such prodrugs lies in the limited data on CYP4Z1’s catalytic properties. Calculating how a prodrug could be activated by a fatty acid hydroxylase enzyme with only the four substrates identified so far is difficult. Thus, more research is required to either broaden the range of CYP4Z1 substrates or to better understand the reaction types produced by CYP4Z1. Another strategy for ovarian cancer treatment is to inhibit CYP4Z1 activity. In contrast to CYP19 (aromatase) [121], CYP4Z1’s functional role in the transformation of normal cells to cancer cells is uncertain, particularly in ovarian cells. Thus, for now, the development of CYP4Z1-activated prodrugs appears to be a more feasible strategy, as abundant CYP4Z1 is available in cancer cells for the successful activation of prodrugs, even though its functional role is unknown. Since all subtypes of ovarian cancer express CYP4Z1, CYP4Z1-activated prodrugs should, in principle, be effective against all subtypes of ovarian cancer and ovarian cancer-derived metastases that have also been proven to express CYP4Z1 [15,20]. Importantly, another therapeutic option for the treatment of ovarian cancer resulted from a recent breakthrough in the detection of generated CYP4Z1 autoantibodies on the surface of ovarian cancer cells [68]. This may lead to novel solutions in the development of successful immunotherapies for this cancer.

Although not specific to ovarian cancer, CYP2S1 expression is significantly higher in ovarian cancer-derived metastases than in primary tumors and healthy tissues [20]. Such a pattern of CYP2S1 expression in metastatic sites makes it a suitable candidate for the development of CYP2S1-directed antimetastatic therapies. However, determining endogenous ligands, and therefore, deorphanizing this enzyme, appears to be essential in understanding its function in ovarian tumorigenesis.

CYP expression in ovarian cancer can be explored not only because of its overexpression in cancer cells but also due to the unique microenvironment whereby cancers occur, even when a CYP exists but is not definitely overexpressed. One of the primary characteristics of the tumor microenvironment that CYP-targeted treatment currently takes advantage of for patient benefit is hypoxia [122]. The discovery of AQ4N (Banoxantrone), a rationally designed prodrug, has emerged from knowledge of the bioreductive ability of CYPs in solid tumors influenced by hypoxic stress. This prodrug is bioactivated under hypoxic conditions to potent topoisomerase II inhibitor (AQ4), mainly by CYP3A4, CYP1B1, CYP1A1, CYP2S1, CYP2B6, and CYP2W1 [123]. As ovarian cancer patients who express these enzymes have a high risk of mortality and metastasis, this strategy offers a real opportunity for the development of CYP-activated bioreductive prodrugs to be used as adjuvant therapy along with standard treatment.

Regardless of the potential of the therapeutic approach utilizing intratumoral CYP expression, several issues should be taken into account. The presence and activity of a certain CYP in a specific tissue should be assessed to reduce off-target effects and to determine whether its levels are sufficient to promote metabolic cytotoxicity with a clinically appropriate response. Further definitive data on the influence of polymorphism on CYP expression and functionality across large patient cohorts are needed. Furthermore, new and better detection techniques are also required to determine CYP selective expression and specific activity in certain patient-derived cancers [4,119].

Finally, gaining a thorough understanding of the expression and functions of CYP is critical for optimizing existing ovarian cancer therapies and for directing current and new initiatives toward the development of novel drugs with enhanced therapeutic potential and fewer adverse effects.

## 8. Methods

### 8.1. Survival Prognosis Analysis of CYP Isoforms

To assess the prognostic value of CYP isoforms in ovarian cancer patients’ survival, the web-based Kaplan–Meier Plotter tool was used [124], which is an online database that is publicly accessible via http://kmplot.com/analysis/index.php?p=service&cancer=liver_rnaseq (accessed on 31 August 2023), and data on gene expression were obtained from individuals participating in TCGA, the Gene Expression Omnibus (GEO), and the European Genome-Phenome Archive (EGA). The database can assess the clinical impacts of 54,000 genes on survival in 21 different cancer types. The program generates Kaplan–Meier survival plots to investigate the impacts of certain gene expression rates on the prognosis of ovarian cancer patients. Here, the median of CYP isoform expression was used to classify all patients into two distinct categories: high expression and low expression.

### 8.2. Immune Infiltration Analysis 

The TIMER2 (tumor immune estimation resource, version 2, http://timer.comp-genomics.org/) web tool was used to investigate the relationship between the expression of CYPs and immune infiltrates in ovarian cancer (accessed on 31 August 2023). Here, CAFs and CD8 + T-cells were selected for analysis. Different types of algorithms, including TIMER, CIBERSORT, MCPCOUNTER, CIBERSORT-ABS, XCELL, QUANTISEQ, and EPIC, were utilized to estimate the immune infiltrations. A Spearman’s rank correlation test was used to calculate partial correlation values and statistical significance (*p*-values) following purity adjustment, and all data were presented on a scatter plot.

### 8.3. Search Criteria

Searches in PubMed, Google Scholar, and the Science Citation Index were carried out to find the references needed for the current review. The search terms utilized were “cytochrome P450”, “inhibitor”, “prodrug”, “xenobiotics”, and lastly, the names of each CYP. These keywords were used together with “ovarian cancer”, “tumor”, and “neoplasm”. Additional references were chosen if they were found to be of personal interest or if they provided extra information. 

## Figures and Tables

**Table 1:** Functions of CYPs and potential CYP-targeted therapies.

CYP Isoform	Functions	Candidate Drug
CYP1A1	Metabolism of xenobiotics, steroids, and drugs	AFP464, Phortress, ICT2700, and AQ4N
CYP1B1	Metabolism of xenobiotics, steroids, and drugs	DMU135, DMAKO-20, Phortress, and ZYC300
CYP2A6	Drug and steroid metabolism	
CYP2B6	Drug and steroid metabolism	
CYP2C8	Drug and steroid metabolism	
CYP2C9	Drug and steroid metabolism	
CYP2J2	Drug and steroid metabolism	Tanshinone IIA, decursin, and C-26
CYP2S1	Drug and steroid metabolism	AQ4N
CYP2U1	Drug and steroid metabolism	
CYP3A4	Drug and steroid (including testosterone) metabolism	
CYP3A5	Drug and steroid (including testosterone) metabolism	
CYP3A7	Drug and steroid (including testosterone) metabolism	
CYP3A43	Drug and steroid (including testosterone) metabolism	
CYP4B1	Arachidonic acid or fatty acid metabolism	4-IPO
CYP4Z1	Arachidonic acid or fatty acid metabolism	1-benzylimidazole
CYP26A1	Retinoic acid hydroxylase	
CYP51A1	Cholesterol biosynthesis	

## Abbreviations

AA	Arachidonic acid
ACSL4	Acyl-CoA synthetase long-chain family member 4
AhR	Aryl hydrocarbon receptor
AP1	Protein 1
CAR	Constitutive androstane receptor
CDC20	Cell division cycle 20 homolog
CREB	CAMP-response element binding
CYP	Cytochrome P450
DAPK1	Death-associated protein kinase-1
EGA	European Genome-Phenome Archive
GEO	Gene Expression Omnibus
GR	Glucocorticoid receptor
20-HETE	20-hydroxyeicosatetraenoic acid
HNF4a	Hepatic nuclear factor-4a
PKC	Protein kinase C
PXR	Pregnane X receptor
RA	Retinoic acid
RXR	Retinoid X receptor
TCGA	The Cancer Genome Atlas
TIMP-2	Tissue inhibitor of metalloproteinases 2
VEGF A	Vascular endothelial growth factor A
VDR	Vitamin D receptor
